# Osteonecrosis development by tooth extraction in zoledronate treated mice is inhibited by active vitamin D analogues, anti-inflammatory agents or antibiotics

**DOI:** 10.1038/s41598-021-03966-6

**Published:** 2022-01-07

**Authors:** Tomoya Soma, Ryotaro Iwasaki, Yuiko Sato, Tami Kobayashi, Eri Ito, Tatsuaki Matsumoto, Atsushi Kimura, Kana Miyamoto, Morio Matsumoto, Masaya Nakamura, Mayu Morita, Seiji Asoda, Hiromasa Kawana, Taneaki Nakagawa, Takeshi Miyamoto

**Affiliations:** 1 Division of Oral and Maxillofacial Surgery, Department of Dentistry and Oral Surgery, Keio University School of Medicine, 35 Shinano-machi, Shinjuku-ku, Tokyo, 160-8582 Japan; 2 Department of Orthopedic Surgery, Keio University School of Medicine, 35 Shinano-machi, Shinjuku-ku, Tokyo, 160-8582 Japan; 3 Department of Advanced Therapy for Musculoskeletal Disorders II, Keio University School of Medicine, 35 Shinano-machi, Shinjuku-ku, Tokyo, 160-8582 Japan; 4 Department of Musculoskeletal Reconstruction and Regeneration Surgery, Keio University School of Medicine, 35 Shinano-machi, Shinjuku-ku, Tokyo, 160-8582 Japan; 5 Institute for Integrated Sports Medicine, Keio University School of Medicine, 35 Shinano-machi, Shinjuku-ku, Tokyo, 160-8582 Japan; 6 Department of Orthopedic Surgery, Kumamoto University, 1-1-Honjo, Chuo-ku, Kumamoto, 860-8556 Japan; 7 Department of Oral and Maxillofacial Implantology, School of Dentistry, Kanagawa Dental University, 82 Inaoka-cho, Yokosuka, Kanagawa 238-8580 Japan

**Keywords:** Experimental models of disease, Calcium and phosphate metabolic disorders

## Abstract

Invasive dental treatment such as tooth extraction following treatment with strong anti-bone resorptive agents, including bisphosphonates and denosumab, reportedly promotes osteonecrosis of the jaw (ONJ) at the extraction site, but strategies to prevent ONJ remain unclear. Here we show that in mice, administration of either active vitamin D analogues, antibiotics or anti-inflammatory agents can prevent ONJ development induced by tooth extraction during treatment with the bisphosphonate zoledronate. Specifically, tooth extraction during treatment with zoledronate induced osteonecrosis in mice, but administration of either 1,25(OH)_2_D_3_ or ED71, both active vitamin D analogues, significantly antagonized osteonecrosis development, even under continuous zoledronate treatment. 1,25(OH)_2_D_3_ or ED71 administration also significantly inhibited osteocyte apoptosis induced by tooth extraction and bisphosphonate treatment. Administration of either active vitamin D analogue significantly inhibited elevation of serum inflammatory cytokine levels in mice in response to injection of lipopolysaccharide, an infection mimetic. Furthermore, administration of either anti-inflammatory or antibiotic reagents significantly blocked ONJ development following tooth extraction and zoledronate treatment. These findings suggest that administration of active vitamin D, anti-inflammatory agents or antibiotics could prevent ONJ development induced by tooth extraction in patients treated with zoledronate.

## Introduction

Osteonecrosis of the jaw (ONJ) is reportedly induced by tooth extraction in patients treated with strong anti-bone resorptive agents, such as bisphosphonates or denosumab, a neutralizing antibody against receptor activator of nuclear factor kappa B ligand (RANKL), which functions in osteoclast differentiation^1,2^. ONJ is rare, but difficult to cure once it occurs, and activity of daily living is severely inhibited^3,4^. Therefore, preventing ONJ development is mandatory. Therefore, preventing ONJ development is mandatory. Either bisphosphonates or denosumab are frequently used to inhibit bone-resorption by osteoclasts or to prevent bone destruction and hypercalcemia in patients with metastatic bone tumors, giant cell tumors or myeloma^5–7^. Such bone destruction and hypercalcemia are induced by accelerated osteoclastic activity, which is inhibited by anti-resorptive agents^8^. Anti-resorptive agents are also used to treat osteoporosis patients, and although rare, ONJ is also reportedly seen in patients with osteoporosis treated with these drugs after tooth extraction^9,10^. Since osteonecrosis occurs in jaw bone at the extraction site but not in other bones, even after systemic administration of anti-resorptive agents, local oral bacterial infection at the tooth extraction site is thought to underlie ONJ development^11^. Indeed, osteonecrosis development is seen in an infectious osteomyelitis animal model or in patients with osteomyelitis^12,13^. However, mechanisms underlying ONJ development after tooth extraction during treatment with anti-bone resorptive agents remain unclear.

Recently we developed an animal model in which ONJ is induced by tooth extraction in mice treated with zoledronate, a bisphosphonate used to treat patients with bone metastasis or osteoporosis^14–16^. Using that model, we previously reported that the inflammatory cytokine storm underlying ONJ development was significantly inhibited by either targeting inflammatory cytokines such as TNFα, IL-6 or IL-1, or treatment with the TNFα inhibitor etanercept or a neutralizing IL-6 antibody^17^. However, such reagents are expensive, and their use in prevention of rare ONJ development is impractical.

Active vitamin D analogues stimulate calcium absorption from intestine^18,19^ and are frequently used to block hypocalcemia in patients undergoing treatment with strong anti-bone resorptive agents^20,21^. These analogues reportedly enhance the ability of bisphosphonates to elevate bone mass in osteoporosis patients^22^. They also antagonize osteoclast differentiation induced by co-treatment with macrophage colony stimulating factor (M-CSF) and RANKL^23^. Thus, active vitamin D analogues are frequently used with strong anti-resorptive agents like bisphosphonates and denosumab to treat patients with metastatic bone tumors or osteoporosis. However, their effect on ONJ development is unknown.

In the current study, we show that inflammatory conditions leading to ONJ and triggered by a combination of tooth extraction and zoledronate treatment are inhibited by administration of active vitamin D analogues. We also demonstrate that ONJ induced by zoledronate and tooth extraction is antagonized by administration of either anti-inflammatory or antibiotic agents. Our data indicates that inflammatory conditions promoted by treatment with strong anti-resorptive agents, tooth extraction and/or infection underlie ONJ development that can be inhibited by administration of either active vitamin D analogues, antibiotics or anti-inflammatory drugs without discontinuation of zoledronate.

## Results

### Administration of active vitamin D analogues inhibits ONJ development induced by combined zoledronate treatment and tooth extraction

**Figure 1:**
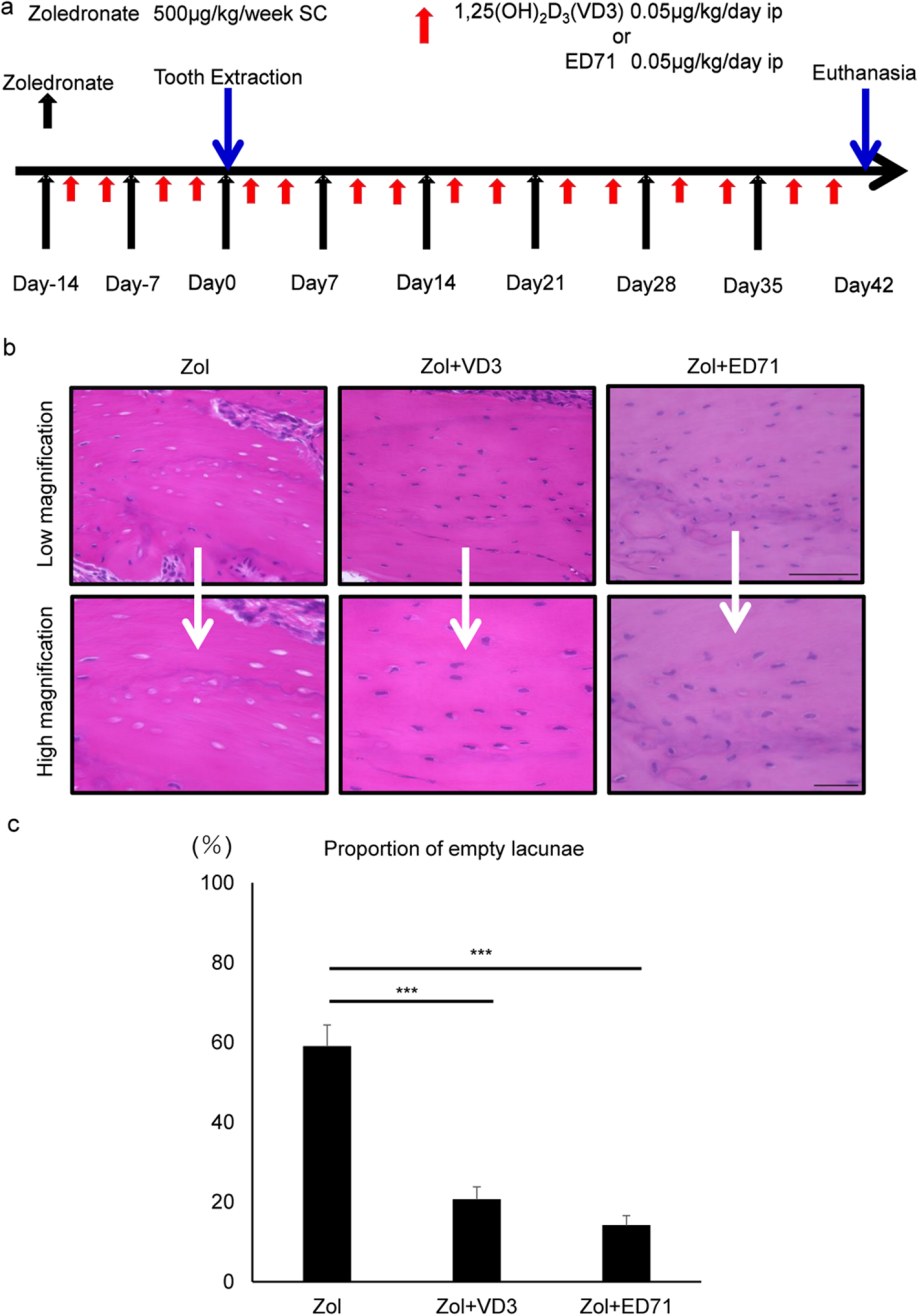
ED71 or VD3 treatment significantly blocks osteonecrosis development in mice treated with zoledronate. (**a**) Experimental protocol. In all experiments, 8-week-old female C57BL/6 mice received subcutaneous injection of zoledronate (500 μg/kg) once a week. Vehicle (ethanol: 0.05 µl/kg/day), ED71 or 1,25(OH)_2_D_3_ (VD3) (0.05 µg/kg/day) was intraperitoneally injected twice a week for 2 weeks before extraction and afterwards twice a week. (**b**) Two weeks after the first injection when mice were 10 weeks old, the right first and second molars in mandible were extracted. Six weeks after extraction, mandibles were removed, stained with HE, observed microscopically, and the percent of empty lacunae among all lacunae was evaluated. Scale bars = 100 μm (upper) and 20 μm (lower) panels. (**c**) Data represents mean relative proportion of empty lacunae among all lacunae in bone ± SD (each with n = 5, ***P < 0.001). Representative data are shown of at least two independent and identical experiments, each with n = 5.

ONJ development in mice is induced by tooth extraction during administration of zoledronate^17,24–26^. Here, we administered zoledronate to wild-type mice once a week for 2 weeks before tooth extraction, and continued treatment on a weekly basis thereafter for 6 weeks (Fig. 1a). In this condition, formation of empty lacunae, a criteria of ONJ, was induced 6 weeks after extraction in jaw bones at the extraction site (Fig. 1b, c). We co-administered either 1,25(OH)_2_D_3_ or ED71, both active vitamin D analogues, with zoledronate (Fig. 1a), and found that empty lacunae formation was significantly inhibited by co-treatment with either drug, without discontinuation of zoledronate treatment (Fig. 1b, c).

### Active vitamin D analogues do not block induction of inflammatory cytokines in osteoclast progenitors by zoledronate or *Porphyromonas gingivalis *in vitro

**Figure 2:**
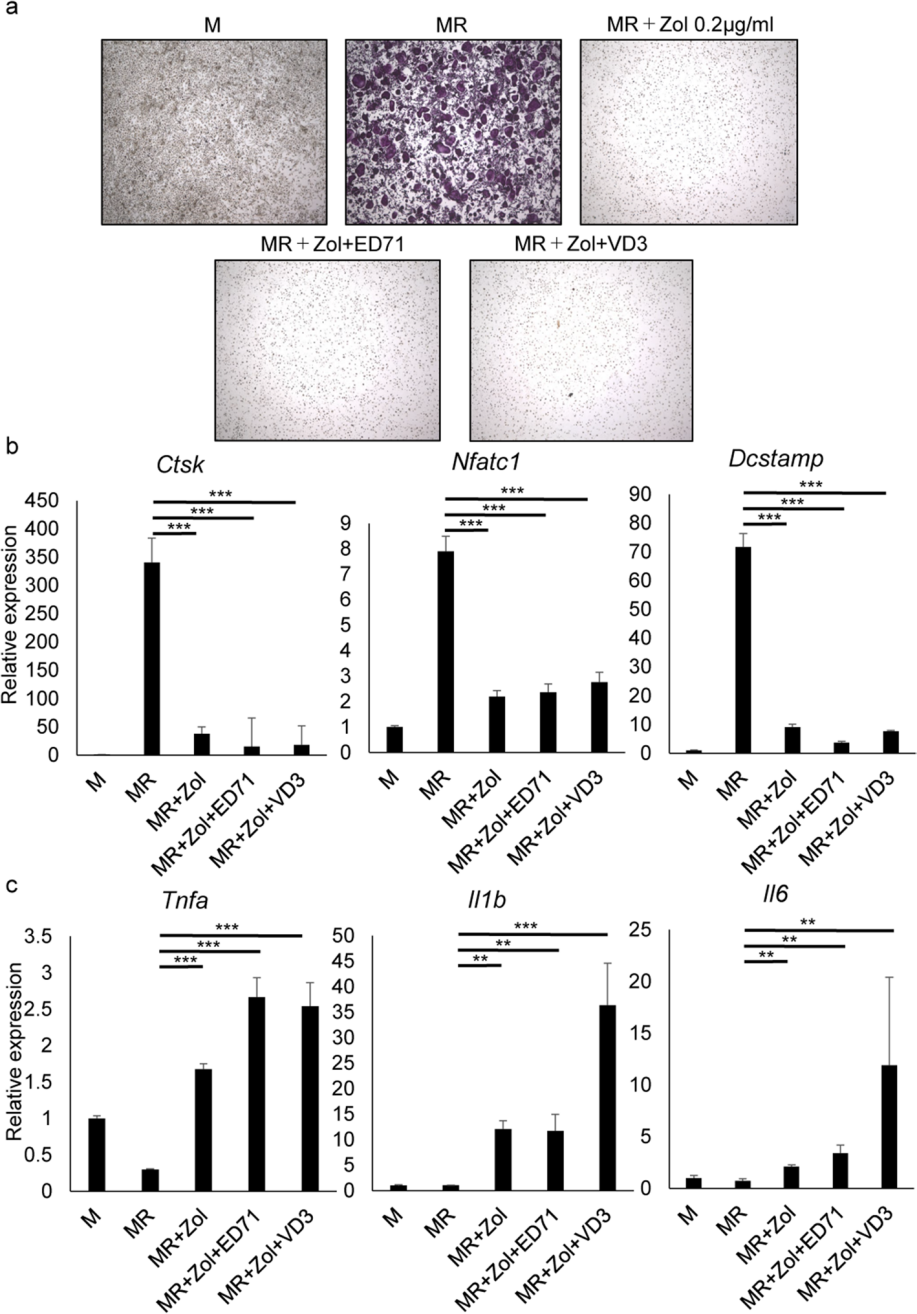
ED71 or VD3 does not inhibit inflammatory cytokine expression in macrophages or osteoclasts. Osteoclast progenitors were isolated from wild-type mice and cultured in the presence or absence of M-CSF (M) or M-CSF (M) and RANKL (R) with or without 0.2 µg/ml zoledronate (Zol) with or without either ED71(10^-6^ M) or VD3 (10^-6^ M). Osteoclast formation was evaluated by TRAP staining (**a**) or quantitative RT-PCR analysis of expression of indicated osteoclast markers. *Tnfa*, *Il1b*, and *Il6* expression was also analyzed by quantitative RT-PCR (**b**, **c**). Data represent mean *Ctsk, Dcstamp, Nfatc1, Tnfa, Il6* or *Il1b* expression relative to *Actb* ± SD (each with n = 3, **P < 0.01; ***P < 0.001 by ANOVA). Representative data of at least two independent experiments are shown.

To determine the roles of active vitamin D analogues in inhibiting ONJ development, we first cultured osteoclast progenitor cells from mouse bone marrow in the presence of M-CSF and RANKL in vitro and observed both formation of multi-nuclear TRAP-positive osteoclasts and significantly upregulated expression of the osteoclastic genes *Cathepsin K* (*Ctsk*), *nuclear factor of activated T cells 1* (*Nfatc1*) and *dendritic cell transmembrane protein* (*Dcstamp*) (Fig. 2a, b). By contrast, M-CSF and RANKL treatment inhibited expression of the inflammatory cytokines *TNFα* (*Tnfa*), *IL-1β* (Il1b) or *IL-6* (*Il6*) in osteoclast progenitors (Fig. 2c). Osteoclastogenesis of M-CSF and RANKL-treated cells was significantly inhibited by zoledronate, and zoledronate-treated cells differentiated into inflammatory cytokine-expressing cells (Fig. 2a–c). Elevation of inflammatory cytokine levels reportedly promotes osteonecrosis development^12,17^. Thus, we asked whether treatment with vitamin D analogues could reverse the zoledronate effects in osteoclast progenitors. However, inhibition of multi-nuclear TRAP-positive osteoclast formation and expression of osteoclastic genes by zoledronate was not rescued in the presence of active vitamin D analogues (Fig. 2a, b). Moreover, expression of inflammatory cytokines was significantly stimulated by zoledronate in osteoclast progenitors and enhanced rather than inhibited by either active vitamin D analogue (Fig. 2c).

**Figure 3:**
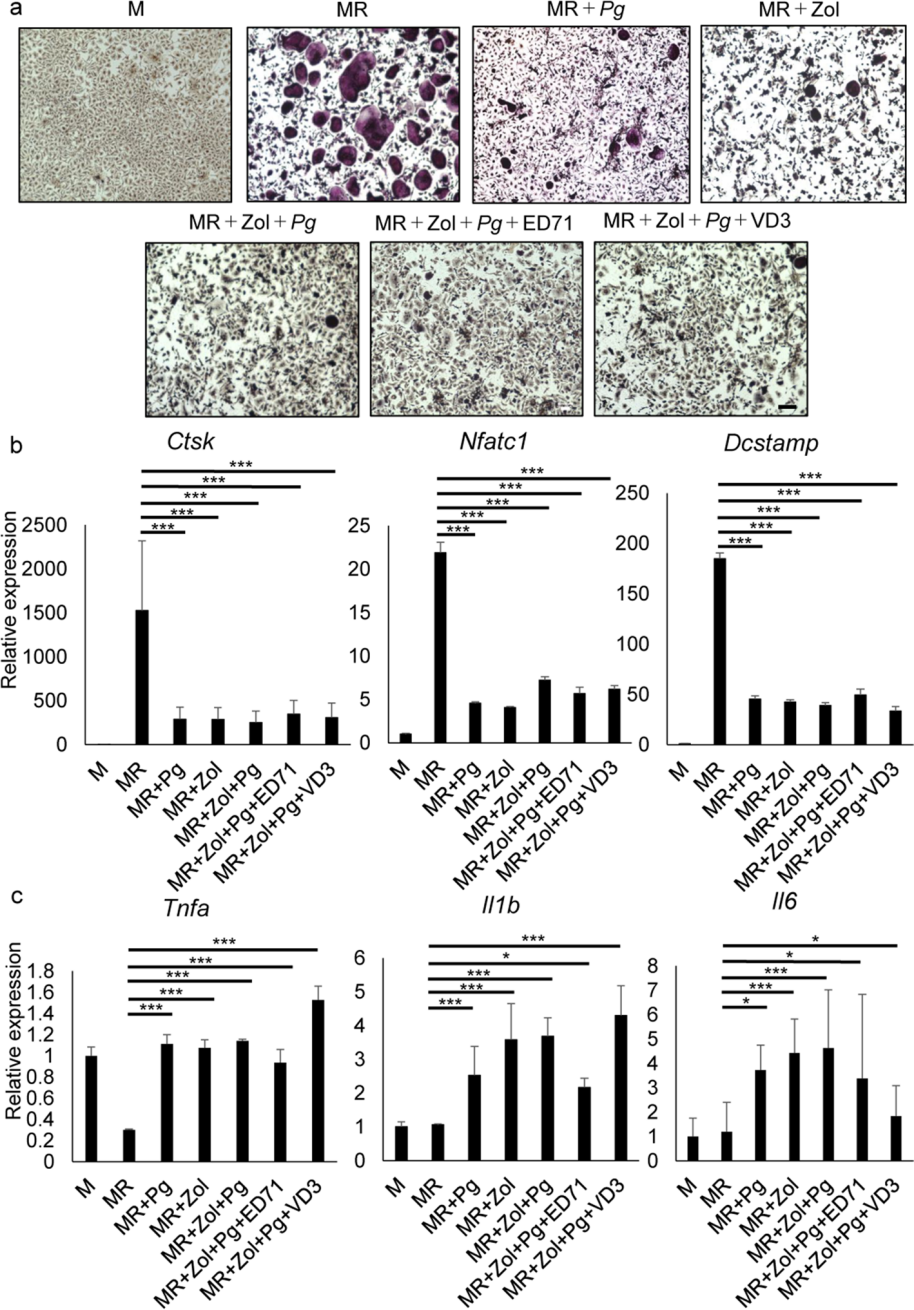
Treatment of osteoclast progenitors with *Porphyromonas gingivalis* extract inhibits osteoclastogenesis and VD3 or ED71 increases inflammatory cytokine expression. Osteoclast progenitors were isolated from wild-type mice and cultured in the presence or absence of M-CSF (M) or M-CSF (M) and RANKL (R) with or without 0.2 μg/ml zoledronate (Zol) with or without either ED71(10^-6^ M) or VD3 (10^-6^ M) and/or *Porphyromonas gingivalis* extract (Pg). Osteoclast formation was evaluated by TRAP staining (**a**) or by quantitative RT-PCR to analyze expression of indicated markers. *Tnfa, Il6, and Il1b* expression in indicated groups of progenitor cells, as evaluated by quantitative RT-PCR (**b**, **c**). Scale bar = 100 μm. Data represent mean indicated transcript levels relative to *Actb* ± SD (each with n = 3, *P < 0.05; ***P < 0.001; NS, not significant, by ANOVA). Representative data are shown of at least two independent and identical experiments each with n = 3.

*Porphyromonas gingivalis* (Pg) is a major oral bacteria and a pathogen in the case of periodontitis^27,28^. Osteoclastogenesis induced by combined M-CSF and RANKL treatment was inhibited by a Pg lysate or zoledronate, and inhibition by either one was not rescued by treatment with an active vitamin D analogue in vitro (Fig. 3a, b). Finally, co-treatment with zoledronate and a Pg lysate induced expression of inflammatory cytokines, and that induction was not blocked by treatment with vitamin D analogues (Fig. 3c).

### Active vitamin D analogues inhibit osteocyte apoptosis induced by zoledronate administration and tooth extraction

**Figure 4:**
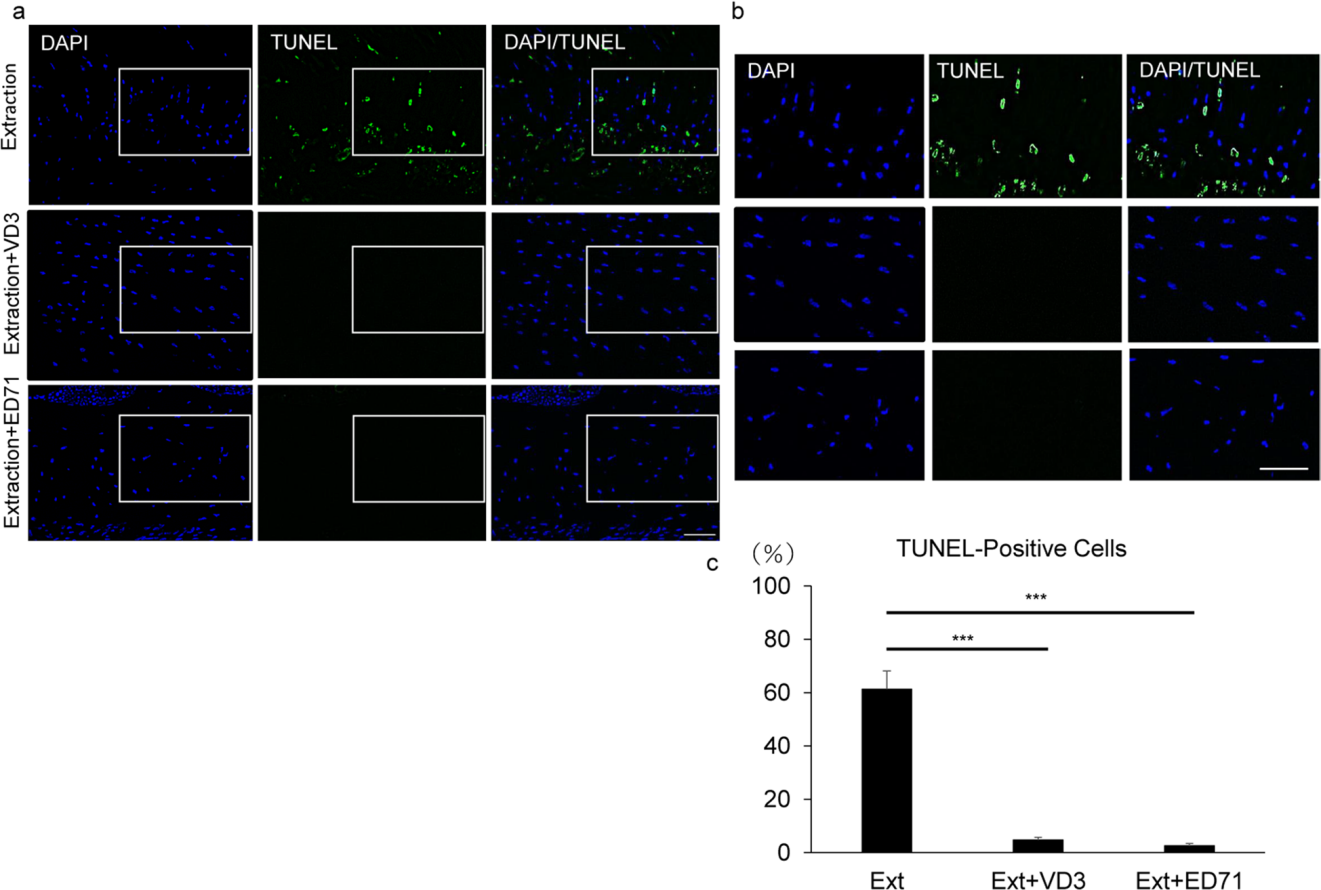
Osteocyte apoptosis is significantly blocked by VD3 and ED71. (**a**, **b**) C57BL/6 mice were administered zoledronate (500 μg/kg) or vehicle (PBS) once a week for 2 weeks and ED71 (0.5 µg/kg) or 1,25(OH)_2_D_3_ (VD3) (0.5 µg/kg) intraperitoneally twice a week and then the right first and second molars in the mandibles were extracted. Four days later, bone sections from mice with zoledronate + tooth extraction (Extraction), VD3 + tooth extraction (Extraction + VD3) or ED71 + Zoledronate + (Zoledronate + ED71) were prepared and labeled with Biotin-dUTP using terminal deoxynucleotidyl transferase (TdT), followed by Avidin-DTAF as TUNEL staining (TUNEL) to identify apoptotic cells. Nuclei were visualized by DAPI. Sections were observed under a fluorescence microscope. (**c**) Data showing mean percentage (%) of TUNEL-positive relative whole osteocytes in bone ± SD (each with n = 5, ***P < 0.001). Representative data are shown of at least two independent and identical experiments, each with n = 5. Bar = 100 µm.

To define mechanisms underlying inhibition of ONJ by vitamin D analogues, we analyzed osteocyte apoptosis using TUNEL staining (Fig. 4). Wild-type mice were administered zoledronate once a week for 2 weeks before tooth extraction. Four days after tooth extraction, osteocyte apoptosis was induced by a combination of zoledronate administration and tooth extraction in mice (Fig. 4). Interestingly, however, osteocyte apoptosis as indicated by TUNEL-positivity was significantly inhibited by co-administration of either active vitamin D analogue in the presence of continuing zoledronate treatment (Fig. 4).

**Figure 5:**
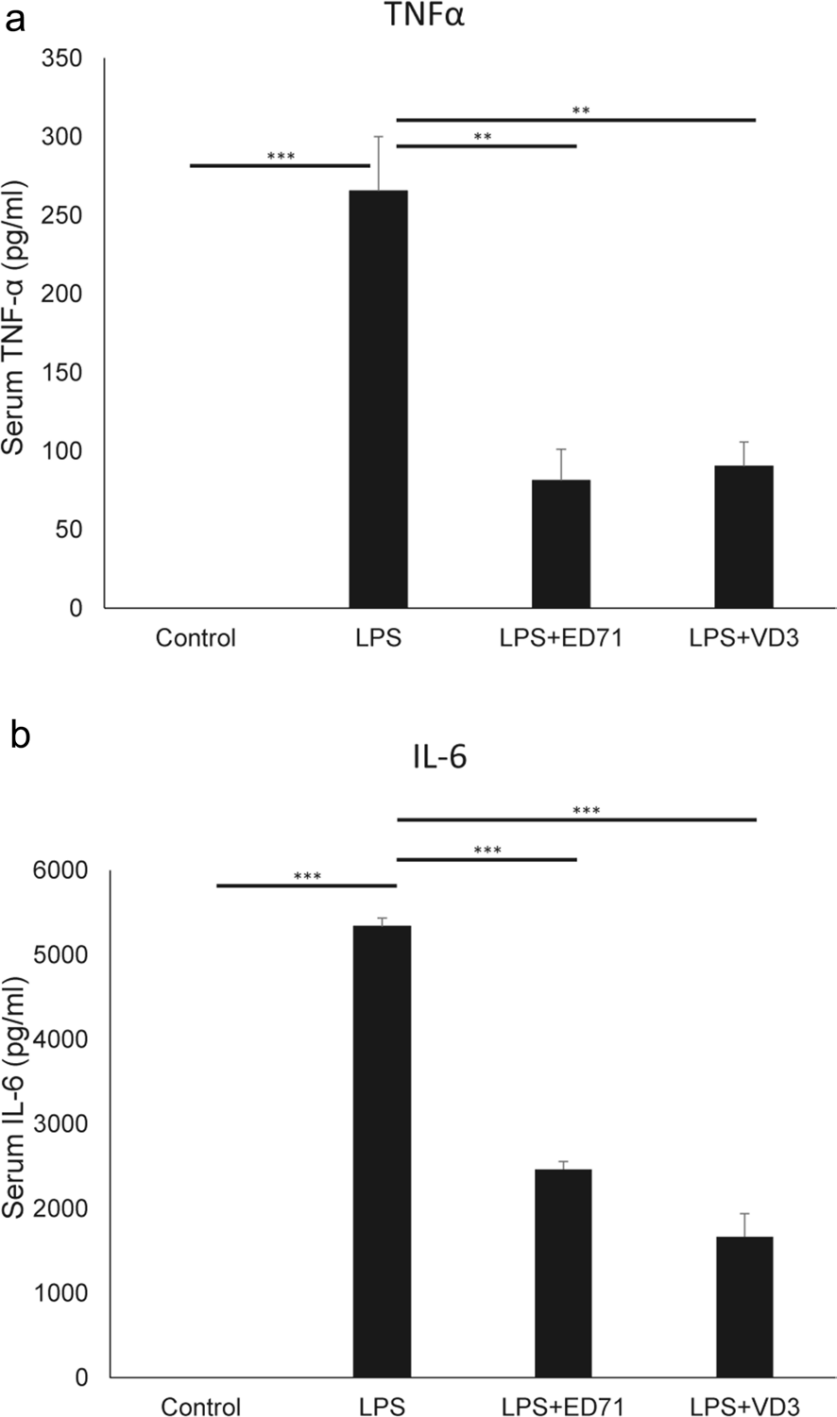
ED71 or VD3 treatment antagonizes induction of inflammatory cytokines by LPS. C57BL/6 mice were intraperitoneal injected with LPS (150 µg/kg). In ED71 + LPS group and VD3 + LPS group, C57BL/6 mice were pretreated with ED71 (0.05 mg/kg) or 1,25(OH)_2_D_3_ (VD3) (0.05 mg/kg) 24 h and 1 h before LPS injection. Maternal serum was collected 2 h after LPS injection. Serum TNF-α (**a**), IL-6 (**b**) were measured using ELISA. All data were expressed as means ± SD of six samples from 5 different mice (n = 5 each, **P < 0.01; ***P < 0.001). Representative data are shown of at least two independent and identical experiments, each with n = 5.

Serum TNFα and IL-6 levels as analyzed by ELISA were significantly elevated two hours after administration of lipopolysaccharide (LPS), a bacterial infection mimetic, to wild-type mice, and such elevation was significantly inhibited by a co-administration of either of the two active vitamin D analogues (Fig. 5a, b). Thus, either analogue has anti-inflammatory activity.

### Administration of either anti-inflammatory drugs or antibiotics inhibits ONJ induced by combined zoledronate treatment and tooth extraction in vivo

**Figure 6:**
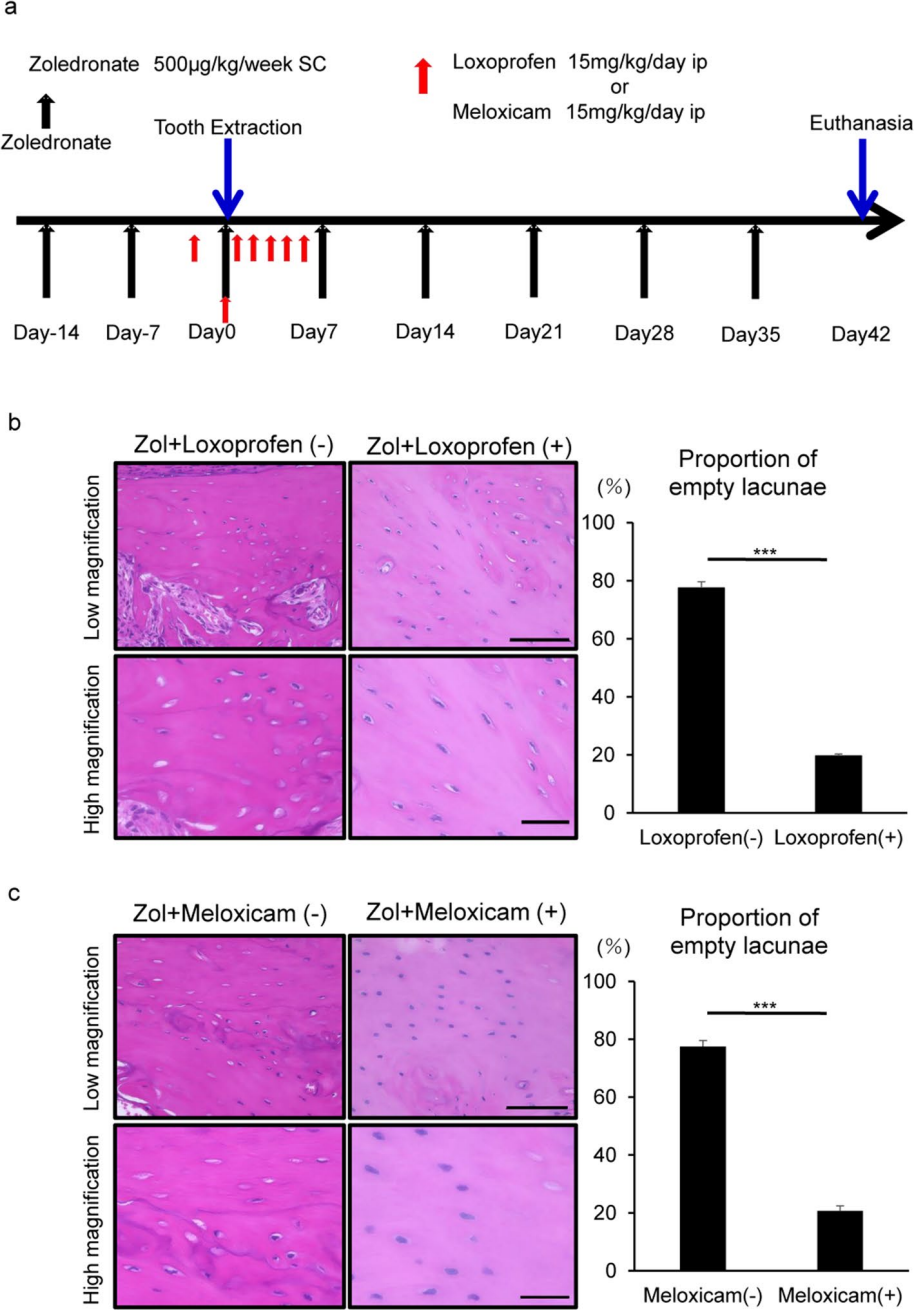
Osteonecrosis induced by tooth extraction and zoledronate administration is significantly blocked by non-steroidal anti-inflammatory drugs. (**a**) Experimental protocol. (**b**, **c**) Loxoprofen sodium hydrate and Meloxicam (15 mg/kg/day), a non-steroidal anti-inflammatory drug, or a Vehicle (control) water was administered by intraperitoneally a day before and 6 days after surgery for a total of 1 week. Six weeks after extraction, alveolar bone sections from the right mandible were prepared and stained with HE, and the proportion of empty lacunae among all lacunae was calculated. Scale bars = 100 (upper) or 20 μm (lower) panels. Data shows mean percentage (%) of empty lacunae ± SD (n = 5 each, ***P < 0.001, by a Mann–Whitney test). Representative data are shown of at least two independent and identical experiments, each with n = 5.

To determine whether elevated inflammatory cytokine levels function in induction of ONJ in vivo, we administered the anti-inflammatory drug loxoprofen intraperitoneally to mice 1 day before tooth extraction and then daily for 6 days (Fig. 6a). Concomitantly, zoledronate was administered once a week for 2 weeks before and 6 weeks after tooth extraction, and then, ONJ development was analyzed (Fig. 6a). ONJ development, as assessed by formation of empty lacunae after tooth extraction, was significantly blocked by loxoprofen administration even in the presence of ongoing zoledronate treatment (Fig. 6b). Similarly, administration of meloxicam, another anti-inflammatory drug (Fig. 6a), significantly blocked ONJ development induced by combined zoledronate and tooth extraction (Fig. 6c).

**Figure 7:**
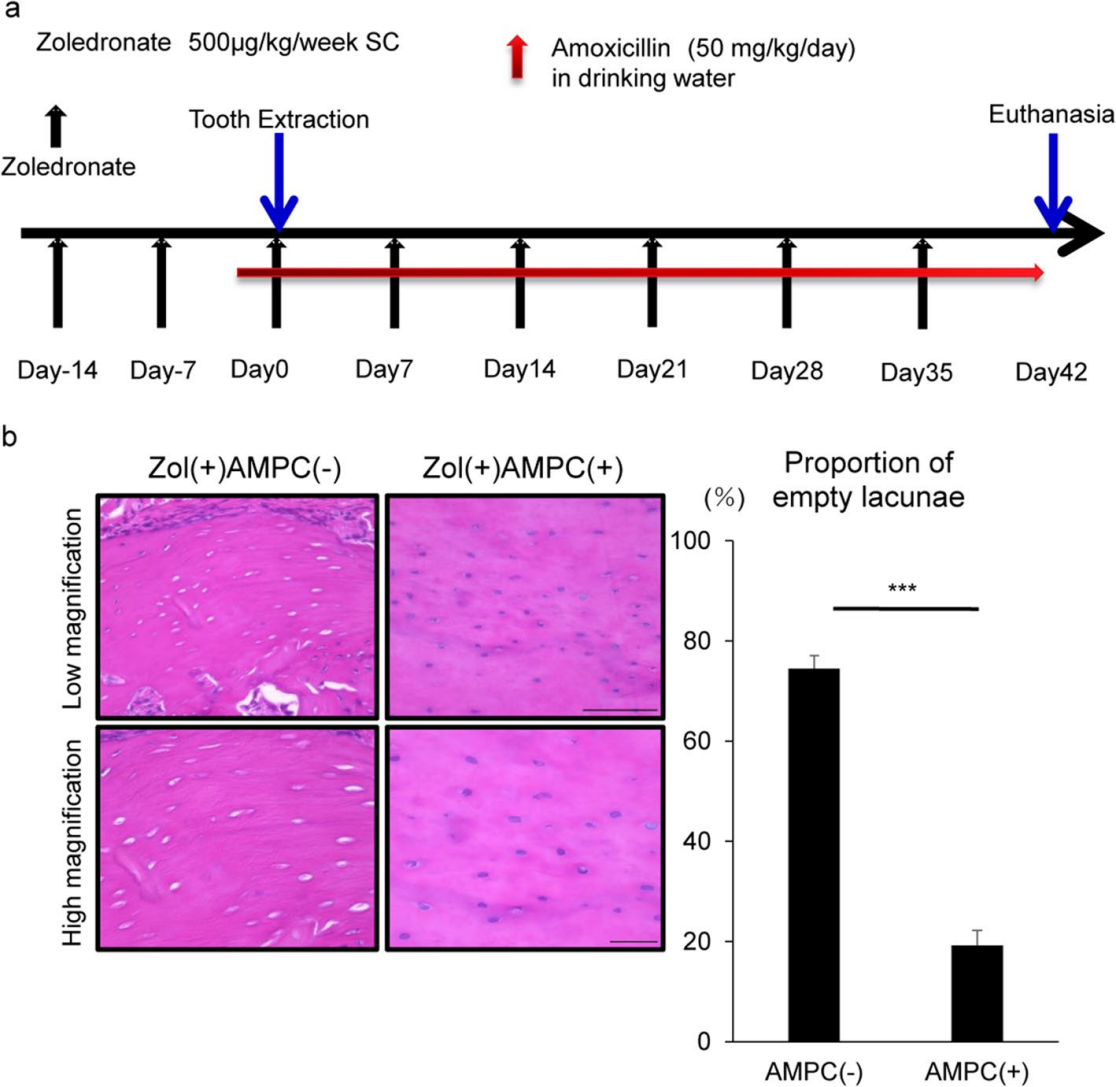
Osteonecrosis induced by tooth extraction and zoledronate administration is significantly blocked by antibacterial drugs. (**a**) Experimental protocol. (**b**) Amoxicillin Hydrate, an antibacterial drug, or normal drinking water was administered orally a day before and 6 weeks after surgery for a total of 6 weeks and 1 day. Six weeks after extraction, alveolar bone sections from the mandible were prepared and stained with HE, and the proportion of empty lacunae among all lacunae was calculated. Scale bars = 100 (upper) or 20 μm (lower) panels. Data shows mean percentage (%) of empty lacunae ± SD (n = 5 each, ***P < 0.001, by a Mann–Whitney test). Representative data are shown of at least two independent and identical experiments, each with n = 5.

Bacterial infection also increases levels of inflammatory cytokines^29,30^, and infectious osteomyelitis reportedly promotes osteocyte apoptosis^12^. Others report the presence of periodontitis, even in mice maintained in specific pathogen-free (SPF) conditions^31^. Thus, we administered the antibiotic Amoxicillin hydrate (AMPC) to mice that treated with zoledronate 1 day before tooth extraction and then continued Amoxicillin administration daily for 6 weeks after extraction, while continuing zoledronate treatment (Fig. 7a). ONJ development, as measured by formation of empty lacunae, was significantly inhibited by AMPC administration 6 weeks after tooth extraction (Fig. 7b).

**Figure 8:**
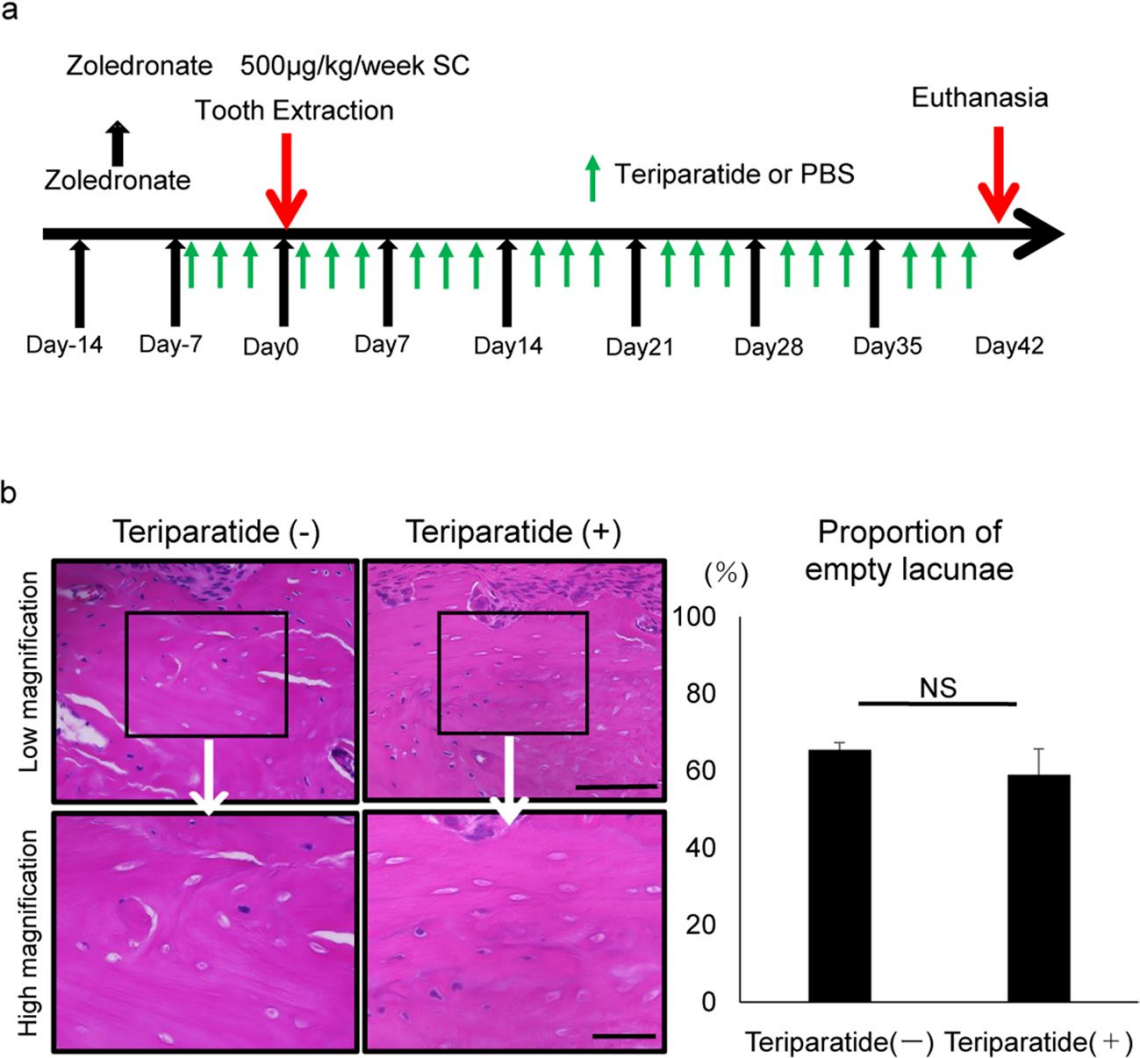
Osteonecrosis development is not effectively blocked by teriparatide. C57BL/6 mice were administered zoledronate for 2 weeks. PTH (80 μg/kg) or PBS was intraperitoneally injected twice a week for 2 weeks before extraction and afterwards twice a week. One week after the first injection when mice were 10 weeks old, the right first and second molars in mandible were extracted. Six weeks after extraction, mandibles were removed, stained with HE, observed microscopically (**a**), and the proportion of empty versus whole lacunae was calculated (**b**). Scale bars = 100 (upper) or 20 μm (lower panels). Data shows the mean percentage (%) of empty versus whole lacunae ± SD (*n* = 5 each, NS, not significant). Representative data are shown of at least two independent and identical experiments, each with n = 5.

A condition known as severely suppressed bone turnover (SSBT) is a reported risk factor for ONJ development^32,33^. Teriparatide, a recombinant parathyroid hormone (1–34), stimulates bone turnover and can reportedly counteract or even reverse ONJ induced by anti-resorptive agents following invasive dental treatment in humans^34^. To analyze the effects of teriparatide in inhibiting ONJ development, teriparatide was administered three times a week from two weeks before and 6 weeks after tooth extraction concomitantly with zoledronate as shown in Fig. 8. However, in our model, teriparatide administration did not inhibit ONJ development induced by zoledronate and tooth extraction (Fig. 8a, b).

Overall, our results suggest that high levels of inflammatory cytokines promote ONJ development following zoledronate treatment and tooth extraction, and that ONJ is inhibited by anti-inflammatory agents, such as active vitamin D analogues, anti-inflammatory drugs or antibiotics.

## Discussion

Although rare, ONJ limits activity of daily living and worsens quality of life. Thus preventive treatments are needed. ONJ develops due to an inflammatory cytokine storm induced by anti-resorptive agents, local infection, tooth extraction or any combination of these conditions^17,35,36^. We show here that ONJ can be prevented in mice by either active vitamin D analogues, anti-inflammatory drugs or antibiotics, all of which inhibit inflammatory conditions.

We previously showed that inflammatory cytokine expression is inhibited by RANKL treatment of cultured osteoclast progenitor cells but stimulated by treatment with anti-resorptive agents or bacterial infection, even in the presence of RANKL^17^. Inflammatory cytokine levels, which likely underlie ONJ development, also increase following invasive dental treatment, such as tooth extraction, or oral bacterial infection^17^. As a result, those cytokine levels likely reach or exceed threshold levels necessary to induce osteocyte apoptosis^17^. Indeed, here we demonstrate that administration of anti-inflammatory drugs or antibiotics inhibits ONJ development induced by zoledronate and tooth extraction. We also demonstrate that active vitamin D analogues inhibit ONJ in this context as well as expression of inflammatory cytokines.

Administration of anti-resorptive agents strongly inhibits bone turnover and can lead to SSBT, a risk factor for ONJ development^32,33^. Administration of a bone turnover stimulator, teriparatide, is reportedly effective in antagonizing or treating ONJ development in humans^34,37^. Teriparatide was also reportedly effective in treating infectious osteomyelitis likely by promoting bone turnover^38^. However, we found that teriparatide treatment in mice did not antagonize ONJ development (Fig. 8), suggesting that it is a cytokine storm rather than inhibition of bone turnover that underlies ONJ development.

Vitamin D has diverse effects on bone biology and anti-inflammation. Active vitamin D analogues are frequently co-administered with anti-osteoporotic drugs such as bisphosphonates, denosumab or romosozumab, and may mobilize calcium to increase bone mass and inhibit hypocalcemia potentially caused by bone mass-increasing agents. Active vitamin D stimulates RANKL expression in osteoblastic cells and promotes osteoclast differentiation^39^. Moreover, active vitamin D analogues reportedly inhibit osteoclastogenesis induced by RANKL by downregulating c-Fos, a transcription factor essential for osteoclast differentiation^40^. Here, we show that inflammatory cytokine expression induced by LPS was significantly inhibited by active vitamin D analogues, and this activity is likely protective against ONJ development. However, a large proportion of people exhibit vitamin D deficiency^41^ and therefore are potentially at risk for ONJ development. Thus, we conclude that for these individuals, taking active vitamin D analogues or vitamin D supplements would be effective in decreasing the risk of ONJ development.

Infectious osteomyelitis is also a risk factor for osteocyte apoptosis^12^. We show that inflammatory cytokine expression was stimulated by infectious conditions in mice. Thus, immediate tooth extraction may be considered preferable to prevent ONJ. Patients treated with strong anti-resorptive agents are occasionally advised to stop taking those agents before and after invasive dental treatment such as tooth extraction due to the risk of ONJ development^42^. However, discontinuation of anti-resorptive agents reportedly reduces bone mass and may increase risk of fragility fractures in patients^43,44^. Our data indicate that ONJ development initiated by zoledronate treatment and tooth extraction is inhibited by either active vitamin D analogues, anti-inflammatory agents or antibiotics, even in the presence of zoledronate treatment.

In conclusion, our data in mice suggest a way to inhibit ONJ development induced by zoledronate and tooth extraction without discontinuation of zoledronate treatment.

## Materials and methods

### Animal model

C57BL/6 background wild-type mice were purchased from Sankyo Labo Service (Tokyo, Japan). Mice were maintained under specific pathogen-free (SPF) conditions in animal facilities certified by the Keio University Institutional Animal Care and Use Committee, and animal protocols were approved by that committee. Mice were housed up to 5 per cage and kept on a 12 h light/dark cycle. Sterile distilled water and a standard diet (CLEA Rodent Diet CE-2, Japan) were available ad libitum. All mouse studies were performed in accordance with Institutional Guidelines on Animal Experimentation at Keio University of The Keio University Institutional Animal Care and Use Committee. Various doses of zoledronate administered to promote ONJ development have been reported in mouse studies: some utilized a single injection at doses from 0.1 to 0.54 mg/kg before tooth extraction^24,25,45,46^, and others administered zoledronate ranging from 0.1 to 0.6 mg/kg/week before and after tooth extraction^17,26,47–55^. Also, zoledronate has been administered to mice either intra-venously^48,52^, intra-peritoneally or subcutaneously^47,49,50,56^. In general, higher doses of zoledronate are administered to mice than to humans^17,26,45,50,56^, as bone turnover in mice is reportedly faster than in humans^57^. Here, we performed experiments using our established ONJ model^17^. Briefly, eight-week old wild-type mice received zoledronate (500 µg/kg) or vehicle (ethanol or PBS) once a week. Two weeks later, when mice were 10 weeks old, the right first and second molars in the mandible were extracted. All mice received a mixture of ketamine (100 mg/ kg) and xylazine (10 mg/kg) by intraperitoneal injection for anesthesia. Groups of mice were administered either ED71 (0.05 µg/kg/day) or 1,25(OH)_2_D_3_ (VD3) (0.05 µg/kg/day) subcutaneously twice a week for 2 weeks before and 6 weeks after extraction. Other mice were administered either Amoxicillin hydrate (50 mg/kg/day) in drinking water or normal water for the week before extraction and continuing for six more weeks. Other mice were administered Loxoprofen sodium hydrate or Meloxicam (15 mg/kg/day), or Vehicle (control) intraperitoneally the day before extraction and then thereafter daily for 6 days. Some mice were injected PTH (80 μg/kg) or PBS intraperitoneally twice a week for 2 weeks before extraction and afterwards twice a week, as previously described^12^. Euthanasia was performed 6 weeks after tooth extraction by cervical dislocation under anesthesia with ketamine hydrochloride (75 mg/kg). No dermatological problems were detected at the site of subcutaneous zoledronate injection (data not shown). Mice that underwent tooth extraction did not exhibit body wight loss (data not shown). All methods are carried out in accordance with the ARRIVE guidelines.

### Chemicals, drugs and reagents

The following reagents were purchased for the study: Lipopolysaccharide (*Escherichia coli* LPS, serotype 0127: B8;Sigma-Aldrich Co., St. Louis, MO, USA); ED71 (Chugai Pharmaceutical CO., LTD. Tokyo, Japan); 1,25(OH)_2_D_3_ (VD3) (Wako Pure Chemicals Industries, Osaka, Japan); Amoxicillin Hydrate (Meiji Holdings Co., Ltd., Tokyo, Japan); Loxoprofen Sodium hydrate and Meloxicam (Tokyo Chemical Industry., Tokyo, Japan); and Teriparatide (Asahi Kasei Pharma Corporation., Tokyo, Japan).

### Histopathological and fluorescent immunohistochemical analysis

Mouse mandibles were removed and decalcified in 10% EDTA, pH7.4, before embedding. At the time of mandible removal, mice exhibited fistulas as pinholes in the mucosa at the site of tooth extraction (Fig. S1), and the jaw bone could be touched through the fistula by a periodontal probe. Paraffin-embedded mandible sections were deparaffinized and rehydrated in a graded ethanol series. Hematoxylin and eosin (HE) staining was performed according to standard methods. For each fluorescent immunohistochemistry assay, sections were subjected to microwave treatment for 10 min in 10 mM citrate buffer solution (pH 6.0) for antigen retrieval, as described^58^. After blocking with 3% BSA in PBS for 1 h, sections were stained using a MEBSTAIN Apoptosis TUNEL Kit Direct (Medical & Biological Laboratories Co., Ltd., Nagoya, Japan). Nuclei were visualized by DAPI (#D1306 1:750; Wako Pure Chemicals Industries, Osaka, Japan). Empty lacunae located just beneath the extracted tooth were detected in H&E-stained mandible sections (Fig. S2). The proportion of empty lacunae was calculated relative to total (empty + non-empty) lacunae.

### In vitro osteoclast formation

Bone marrow cells isolated from mice femurs and tibias were cultured 72 h in α-MEM (Sigma-Aldrich, St. Louis, MO) containing 10% heat-inactivated fetal bovine serum (FBS) (SAFC Biosciences) and GlutaMax (Invitrogen, Carlsbad, CA) supplemented with M-CSF (50 ng/mL, Kyowa Hakko Kirin Co., Tokyo, Japan). Subsequently, adherent cells were collected and cultured 4 days in 96-well plates (1 × 10^5^ cells per well) under indicated conditions containing M-CSF (50 ng/mL) and recombinant soluble RANKL (25 ng/mL, PeproTech Ltd., Rocky Hill, NJ), with or without ED71 (Chugai Pharmaceutical Co., Ltd, 10^–6^ M), 1,25(OH)_2_D_3_ (Wako Pure Chemicals Industries, 10^-6^ M), or 1.0% *Porphyromonas gingivalis strain W83 (Pg)* extract (prepared by incubating 3.0 × 10^10^ CFU *Pg* in 100 μl RIPA buffer (1% Triton X-100, 1% sodium deoxycholate, 0.1% SDS, 150 mM NaCl, 5 mM EDTA, 1 mM dithiothreitol, 10 mM Tris–HCl, pH7.5)) and supplemented with a protease inhibitor cocktail (Sigma-Aldrich, St. Louis, MO) and MG-132 (EMD Millipore Corporation). Cell lysates were prepared using RIPA buffer supplemented with the protease inhibitor cocktail and MG-132. Medium was changed every 2 days. Osteoclastogenesis was evaluated based on tartrate-resistant acid phosphatase (TRAP) staining, as described^59,60^.

### Quantitative real-time PCR

Samples were directly treated with TRIzol reagent (Invitrogen, Carlsbad, CA). Total RNA was isolated from bone marrow cultures using a RNeasy mini kit (QIAGEN, Antwerp, Belgium) and stored at − 80 °C before use. First-stranded cDNA synthesis was performed using oligo (dT) primers and reverse transcriptase (Wako Pure Chemicals Industries), as described^61,62^. Quantitative RT-PCR was performed using SYBR Premix ExTaq II reagent and a DICE thermal Cycler Real Time System III (Takara Bio Inc., Shiga, Japan), according to the manufacturer’s instructions. *β-actin* (*Actb*) expression served as an internal control. Primers for *Actb, Ctsk, Nfatc1, Dcstamp, Tnfa, Il1b and Il6* were as follows:

- *β-actin*-forward: 5′-TGAGAGGGAAATCGTGCGTGAC-3′
- *β-actin*-reverse: 5′-AAGAAGGAAGGCTGGAAAAGAG-3′
- *Ctsk*-forward: 5′-ACGGAGGCATTGACTCTGAAGATG-3′
- *Ctsk*-reverse: 5′-GGAAGCACCAACGAGAGGAGAAAT-3′
- *Nfatc1*-forward: 5′-CAAGTCTCACCACAGGGCTCACTA-3′
- *Nfatc1*-reverse: 5′-GCGTGAGAGGTTCATTCTCCAAGT-3′
- *Dcstamp-forward: 5′-TCCTCCATGAACAAACAGTTCCAA-3′*
- *Dcstamp-reverse: 5′-AGACGTGGTTTAGGAATGCAGCTC-3′*
- *Tnfa*-forward: 5′-AAGCCTGTAGCCCACGTCGT-3′
- *Tnfa*-reverse: 5′-GGCACCACTAGTTGGTTGTCTTTG -3′
- *Il1b*-forward: 5′-AAGTTGACGGACCCCAAAAGAT-3′
- *Il1b*-reverse: 5′-AGCTCTTGTTGATGTGCTGCTG-3′
- *Il6*-forward: 5′-GTCCTTAGCCACTCCTTCTG-3′
- *Il6*-reverse: 5′-CAAAGCCAGAGTCCTTCAGAG-3′

### Enzyme-linked immunosorbent assay (ELISA)

ELISA kits (R&D Systems, Minneapolis, MN, USA) were used to measure mouse TNF-α and IL-6 in maternal sera according to the manufacturer’s protocol and using a multiple plate analyzer (Cytation 5, BioTek Instruments, Inc., Vermont, US).

### Statistical analysis

All quantified data were expressed as means ± SD. Statistical significance of differences between groups was evaluated using Student’s t test, a Mann–Whitney *U* test or a one-way analysis of variance (ANOVA) using statistical software (version 25; SPSS Inc., Chicago, IL, USA) (*P < 0.05; **P < 0.01; ***P < 0.001; NS, not significant, throughout the paper).

## Supplementary Information

Supplementary Information.
