# Supplementary material for: Osteonecrosis development by tooth extraction in zoledronate treated mice is inhibited by active vitamin D analogues, anti-inflammatory agents or antibiotics
Source: Sci Rep. 2022 Jan 7;12:19. doi: 10.1038/s41598-021-03966-6 (PMC8742126; doi:10.1038/s41598-021-03966-6)
Supplement: Supplementary file 1 — Supplementary Information. [file 41598_2021_3966_MOESM1_ESM.pdf]

Osteonecrosis development by tooth extraction in zoledronate treated mice is inhibited by active vitamin D analogues, anti-inflammatory agents or antibiotics

Tomoya Soma[1], Ryotaro Iwasaki[1], Yuiko Sato[2,3], Tami Kobayashi[2,4], Eri Ito[5], Tatsuaki Matsumoto[2], Atsushi Kimura[2], Kana Miyamoto[6], Morio Matsumoto[2], Masaya Nakamura[2], Mayu Morita[1], Seiji Asoda[1], Hiromasa Kawana[1, 7], Taneaki Nakagawa[1] and Takeshi Miyamoto[2, 3, 4, 6]

[1]Division of Oral and Maxillofacial surgery, Department of Dentistry and Oral Surgery, [2]Department of Orthopedic Surgery, [3]Department of Advanced Therapy for Musculoskeletal Disorders II, [4]Department of Musculoskeletal Reconstruction and Regeneration Surgery, [5]Institute for Integrated Sports Medicine, Keio University School of Medicine, 35 Shinano-machi, Shinjuku-ku, Tokyo 160-8582, Japan, [6]Department of Orthopedic Surgery, Kumamoto University, 1-1- Honjo, Chuo-ku, Kumamoto 860-8556, Japan, [7]Department of Oral and Maxillofacial Implantology, School of Dentistry, Kanagawa Dental University, 82 Inaoka-cho, Yokosuka, Kanagawa, 238-8580, Japan.

Running title: *Active vitamin D analogues inhibit osteonecrosis*

Correspondence should be addressed to: T. M., Department of Orthopedic Surgery, Keio University School of Medicine, 35 Shinano-machi, Shinjuku-ku, Tokyo 160-8582, Japan or Department of Orthopedic Surgery, Kumamoto University, 1-1- Honjo, Chuo-ku, Kumamoto 860-8556, Japan

TEL: 81-3-5363-3812, FAX: 81-3-3353-6597, e-mail: miyamoto@z5.keio.jp or miyamoto.takeshi@kuh.kumamoto-u.ac.jp

Figure S1. Macroscopic findings relevant to tooth-extracted mice.

Eight-week-old female C57BL/6 mice received subcutaneous injection of zoledronate (500 μg/kg) once a week. Two weeks after the first injection when mice were ten weeks old, the right first and second molars in the mandible were extracted. Six weeks after extraction, extracted sites were observed macroscopically. The site inside the orange box in the left panel is shown at higher magnification in the right panel. Fistula formation was observed at the site of tooth extraction (marked by yellow arrows).

Figure S2. Site of scoring of empty lacunae.

(left) Schematic showing the site of the extracted first and second molars. (right) Microscopic view of the site scored for empty lacunae following HE staining.

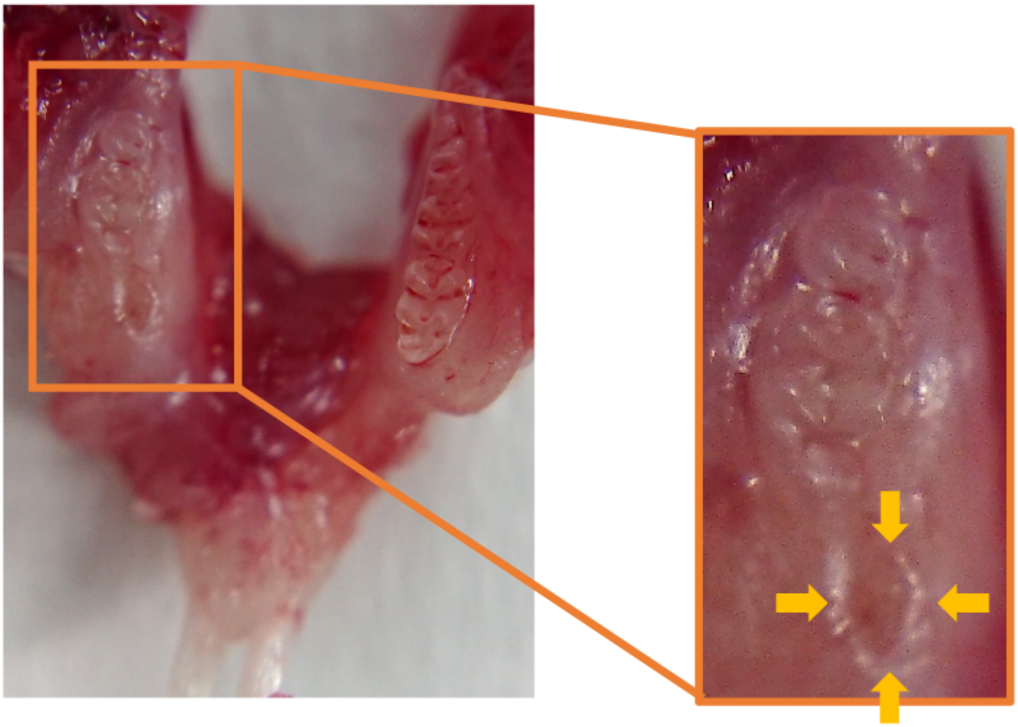

Figure S1 Soma T et al

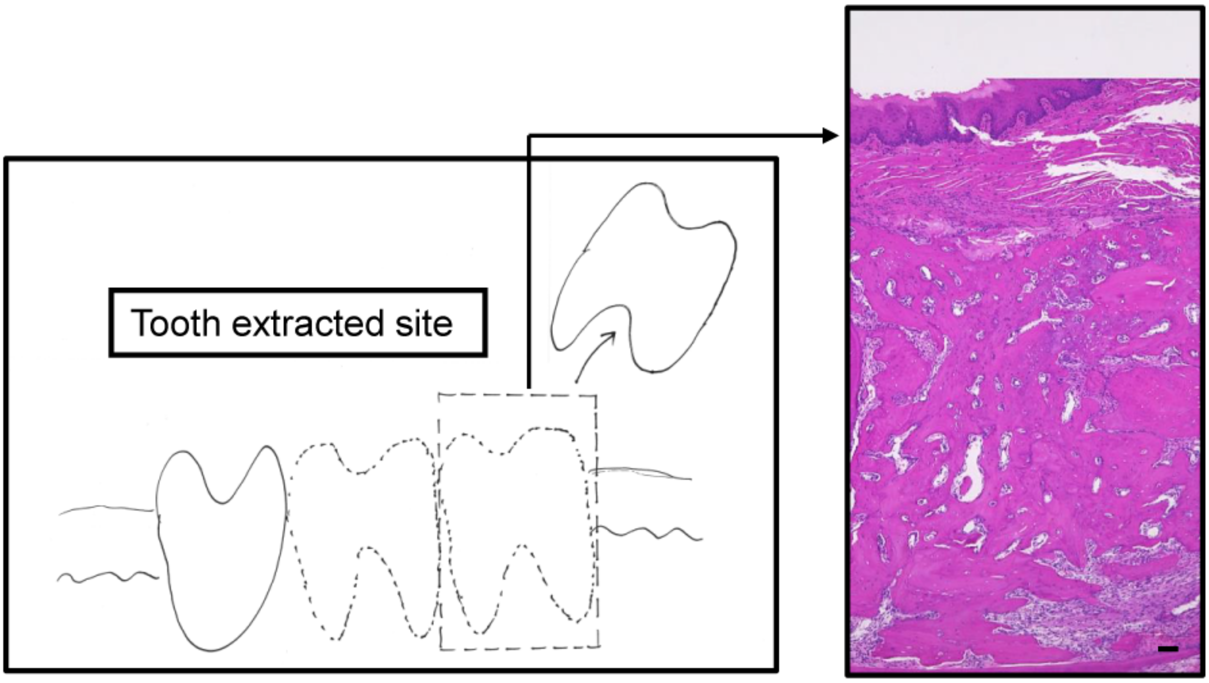

Figure S2 Soma T et al
